# A functional BH3 domain in an aquaporin from *Leishmania infantum*

**DOI:** 10.1038/cddiscovery.2016.43

**Published:** 2016-07-04

**Authors:** C M Genes, H de Lucio, V M González, P A Sánchez-Murcia, E Rico, F Gago, N Fasel, A Jiménez-Ruiz

**Affiliations:** 1 Departamento de Biología de Sistemas, Universidad de Alcalá, Alcalá de Henares 28805, Spain; 2 Laboratory of aptamers, Departamento de Bioquímica-Investigación, IRYCIS-Hospital Ramón y Cajal, Madrid, Spain; 3 Departamento de Ciencias Biomédicas, Universidad de Alcalá, Facultad de Medicina, Alcalá de Henares 28805, Spain; 4 Department of Biochemistry, University of Lausanne, 155 Chemin des Boveresses, Epalinges 1066, Switzerland

## Abstract

Despite the absence of sequences showing significant similarity to any of the members of the Bcl-2 family of proteins in protozoa, experiments carried out in yeast or trypanosomatids have demonstrated that ectopic expression of some of these members alters their response to different death stimuli. Because the BH3 domain is the smallest common signature in all the proteins of this family of apoptosis regulators and also because they are essential for molecular interactions between antagonistic members, we looked for sequences with significant similarity to the BH3 motif in the *Leishmania infantum* genome. Among the top scoring ones, we found the MYLALQNLGDEV amino-acid stretch at the C terminus of a previously described aquaporin, now renamed as Li-BH3AQP. This motif is highly conserved in homologous proteins from other species of the *Leishmania* genus. The association of Li-BH3AQP with human Bcl-X_L_ was demonstrated by both co-immunoprecipitation and yeast two-hybrid experiments. Ectopic expression of Li-BH3AQP reduced viability of HeLa cells and this deleterious effect was abrogated by the simultaneous overexpression of Bcl-X_L_. Although we were not able to demonstrate a reduction in parasite viability when the protein was overexpressed in *Leishmania* promastigotes, a prodeath effect could be observed when the parasites overexpressing Li-BH3AQP were treated with staurosporine or antimycin A. Surprisingly, these parasites were more resistant, compared with wild-type parasites, to hypotonic stress or nutrient deprivation. The prodeath activity was abolished upon replacement of two highly conserved amino acids in this BH3 domain. Taken together, these results point to Li-BH3AQP as the first non-enzymatic protein ever described in trypanosomatids that is involved in cell death.

Research in the field of cell death processes in unicellular parasites displaying features normally associated with apoptosis in metazoan organisms has generated a significant body of literature since the first description in 1996.^1,2^ Biochemical and morphological features resembling this cell death process have been described across yeasts,^3^ algae^4^ and the genera *Trypanosoma*,^1,2,5^ *Leishmania*^6,7^ and *Plasmodium*.^8–11^ The occurrence of apoptosis in unicellular organisms is a challenge for evolutionary theory, as it seems to argue against the Darwinian notion of ‘survival of the fittest’. However, promoting the reproductive success of relatives with a very similar genetic content, such as those resulting from infections of the host with a very low number of parasites, may be a successful strategy to ensure transmission of copies of their genes to future generations.^12^ Thus, programmed cell death (PCD) processes might be useful to regulate parasite density inside the host and aid in avoiding inflammatory responses from the immune system, thereby facilitating a sustained infection.^13^ Nevertheless, the almost absolute absence of dedicated molecular pathways that coordinate this process in single-celled organisms is still a major concern that hampers acceptance of the existence of PCD in parasites.^14^ With the exception of endonucleases G^15–17^ and metacaspases,^18–20^ no other molecule has been implicated in PCD in protozoans.

In this scenario, it is conspicuous that, with a single exception, no coding sequence for any member of the Bcl-2 family has been found to date in the genomic databases of protozoa. It is therefore intriguing that mammalian Bcl-2 proteins can inhibit (e.g. Bcl-X_L_) or induce (e.g. Bax) cell death processes when expressed in yeast,^21,22^ or *Leishmania* cells.^7,23^ Up till now, the yeast proapoptotic BH3-only protein Ybh3p is the unique Bcl-2 family member described in the literature for unicellular organisms. Overexpression of Ybh3p facilitates apoptosis induced by a variety of stimuli, whereas its ablation inhibits death and increases replicative lifespan.^24^ Similar to other mammalian BH3-only proteins, Ybh3p translocates to mitochondria during apoptosis, triggering their depolarization and cytochrome *c* release.

Bcl-2-related proteins share homology in one or several of the four regions designated as the Bcl-2 homology (BH) domains BH1, BH2, BH3 and BH4. Among them, only BH3 is consistently found in all members of the family, as it is essential for binding associations between pro- and antiapoptotic members. Owing to the demonstrated effect of ectopic expression of members of the mammalian Bcl-2 family of proteins in *Leishmania* parasites, we initiated a search for putative BH3-coding sequences in the *Leishmania infantum* genome. This strategy allowed us to identify a BH3 domain in a protein previously annotated as an aquaporin. We then examined the ability of this protein to bind to Bcl-X_L_ and its effects on viability of both HeLa cells and *L. infantum* promastigotes.

## Results

### BH3 domains in *L. infantum*

**Figure 1:**
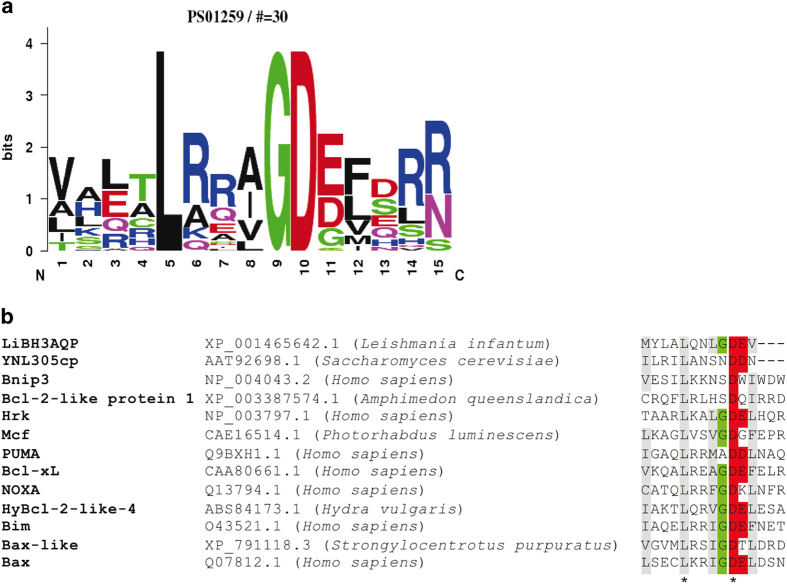
The Li-BH3AQP C-terminal region matches the BH3 consensus pattern. (**a**) Weblogo of the PS01259 pattern highlighting the strongly conserved residues leucine (L), aspartic acid (D) and glycine (G) in positions 5, 9 and 10, respectively. (**b**) Alignment of Li-BH3AQP and representative BH3 domain sequences using the Clustal W software. Colored residues show highly conserved positions in the PS01259 pattern. Gray, hydrophobic residues; green, small residue (G); red, acidic residues.

Previous results from our laboratory demonstrated that ectopic expression of human Bcl-X_L_ in *L. infantum* reduces the death rate of the parasites in response to heat shock.^7^ In an attempt to identify putative proteins able to interact with this antiapoptotic protein, we developed a bioinformatics search for DNA sequences coding for putative BH3 domains in the *L. infantum* genome. The PHI-BLAST (Pattern Hit Initiated BLAST) algorithm was chosen to look for non-redundant protein sequences in the *Leishmania* genus using the central region from the PS01259 motif signature (L-[KARQ]-x-[IVAL]-G-D-[DESG]-[LIMFV]; residues 5–12) as the search pattern. This region contains the most highly conserved residues in the BH3 domain. As a result, we were able to identify the sequence MYLALQNLGDEV (amino acids that fit the search pattern are underlined) at the C terminus of an *L. infantum* protein previously named AQP putative (XP_001465642.1).^25^ This putative BH3 domain is completely conserved in orthologs from *L. donovani* (ACZ51352), *L. major* (XP_001683293), *L. mexicana* (XP_003875596), *L. braziliensis* (XP_001568467) and *L. panamensis* (XP_010702776). The sequence logo^26^ for ProSite entry PS01259 and a multiple sequence alignment of the putative BH3 domain from XP_001465642 and equivalent domains from different members of the Bcl-2 family of proteins are shown in Figure 1. It can be observed that the consensus is good for all residues with the exceptions of (i) a methionine in position 1, which is usually occupied by another hydrophobic amino acid, and (ii) the lack of residues 13–15 because the valine in position 12 in this motif corresponds to the C-terminal amino acid of XP_001465642. Analysis of the sequences of members of this family of proteins in higher eukaryotes reveals that very few of them match the PS01259 pattern exactly. In fact, the recall rate (true positives/(true positives+false negatives)) is as low as 44%,^27^ which reveals the absence of an absolute consensus sequence for the BH3 domain.

XP_001465642.1 (in common to all its orthologs in other *Leishmania* species) belongs to the major intrinsic protein family of proteins, which contain characteristic pore structures that are able to span the plasma membrane and allow the transport of water or small neutral solutes such as glycerol. Because of the presence of a putative BH3 domain, we propose to rename XP_001465642.1 to Li-BH3AQP.

### Structural analysis of Li-BH3AQP

**Figure 2:**
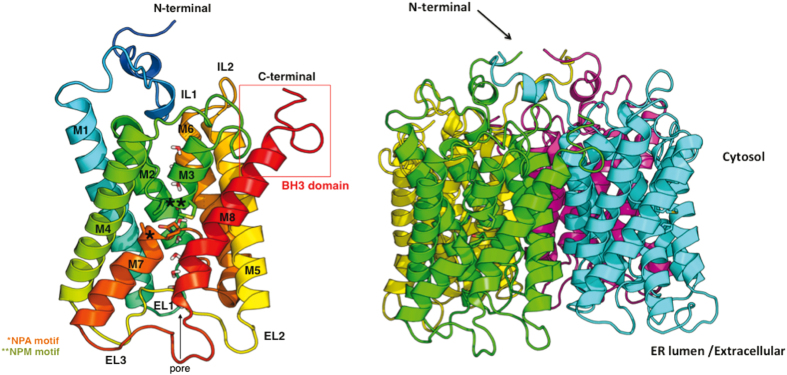
Homology model of monomeric (left) and tetrameric (right) Li-BH3AQP. Water molecules in the channel are shown as sticks. The putative BH3 domain is boxed. * indicates NPA aquaporin signature motif. ** indicates NPM motif.

The 3D structure of this 31 kDa protein (294 residues long) was modeled using the crystal structures of aquaporins Aqy1 from *Pichia pastoris* (Pp-Aqy1, PDB entry 2W2E, 26% sequence identity;^28^ Supplementary Figure S1) and AqpM from *Methanothermobacter marburgensis* (Mm-AqpM, PDB entry 2F2B, 29% sequence identity;^29^ Supplementary Figure S1) as templates. Each monomer in Li-BH3AQP is likely to be formed by six full membrane-spanning helices (M1, M2, M4, M5, M6 and M8), two intracellular loops (IL1 and IL2) defined by residues 130–141 and 207–211, respectively, and three extracellular loops made up of residues 91–95 (EL1), 167–182 (EL2) and 252–264 (EL3) (Figure 2). In addition, each monomer presents two half-membrane-spanning helices M3 (residues 121–129) and M7 (240–249), which constitute a seventh pseudotransmembrane segment that contains the Asn-Pro-Ala (NPA) aquaporin signature motif near the center of the water channel.^30^ It is noteworthy than in the second motif that the Ala residue has been replaced by Met in Li-BH3AQP. The nonpolar side chains of these aliphatic residues have been shown to participate in preserving the orientation of the two asparagines in the center of the channel that are responsible for substrate selectivity^31^ (Figure 2). The Li-BH3AQP loops IL1, EL2 and EL3 present insertions of up to seven residues compared with Pp-Aqy1 or Mm-AqpM, and, according to our model, Cys251 and Cys260 located on EL3 are close enough to make a disulfide bond between them. Regarding the quaternary structure of the protein, Li-BH3AQP would be organized as a homotetramer within the lipid membrane with both N and C termini oriented towards the cytosol. The putative BH3 domain (residues 283-MYLALQNLGDEV-294), which is predicted to be structured as an *α*-helix except for the last four residues, is projected out towards the opposite face of the channel. In such a conformation, it could be accessible to Bcl-2-like proteins, although a portion of it might be partially embedded in the lipid membrane.

**Figure 3:**
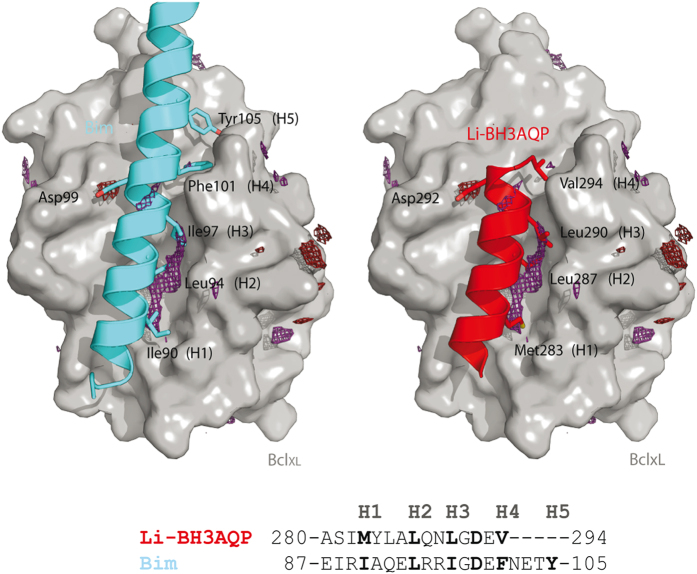
Interaction of Bim BH3 domain (left, cyan) and Li-BH3AQP C terminus (*right*, red) with Bcl-X_L_ (gray surface). Note the correspondence between the calculated affinity maps (dark purple, hydrophobic; red, carbonyl oxygen) on the surface of Bcl-X_L_ and similarly spaced amino-acid side chains from Bim and Li-BH3AQP BH3 domains. H1 to H4 hydrophobic positions in Bim BH3 domain are strictly conserved in Li-BH3AQP.

To assess whether the putative BH3 domain could fulfill the structural features required to interact with Bcl-X_L_, we analyzed our molecular model of Li-BH3AQP with an in-house software named cGRILL,^32^ which is formally similar to Goodford's program GRID.^33^ cGRILL allows the identification of 3D affinity maps between the protein and a number of probes (i.e. CH3, OH, etc.), therefore facilitating the interpretation of protein–protein energetics. As a test case for validation of the method, we chose the murine Bim fragment (residues 83–115) in complex with Bcl-X_L_ (PDB i.d. 1PQ1).^34^ In this experimental structure, it can be seen that the side chains of four hydrophobic residues (H1–H4), namely, Ile90, Leu94, Ile97 and Phe101, each separated by a helical turn, are buried inside four well-defined pockets in Bcl-X_L_. These regions are detected by the cGRILL hydrophobic probe as favorable for binding; the corresponding energy contours are displayed as a dark purple mesh in Figure 3. In addition, Bim orientates Tyr105 towards the less hydrophobic pocket H5. Another distinct region displaying favorable hydrogen-bonding properties is highlighted by the carbonyl oxygen probe whose energy contour (red mesh in Figure 3) overlaps the side-chain carboxylate of Asp99. Our molecular model of the putative Bcl-X_L_: Li-BH3AQP complex shows the hydrophobic side chains of Met283, Leu287, Leu290 and Val294 in the 283-MYLALQNLGDEV-294 stretch matching regions H1–H4 (underlined) in a similar manner to those of Ile90, Leu94 Ile97 and Phe101 in Bim, respectively, whereas the carboxylate of Asp292 occupies the same position as that of Asp99 of Bim.

### Li-BH3AQP binds to Bcl-X_L_

**Figure 4:**
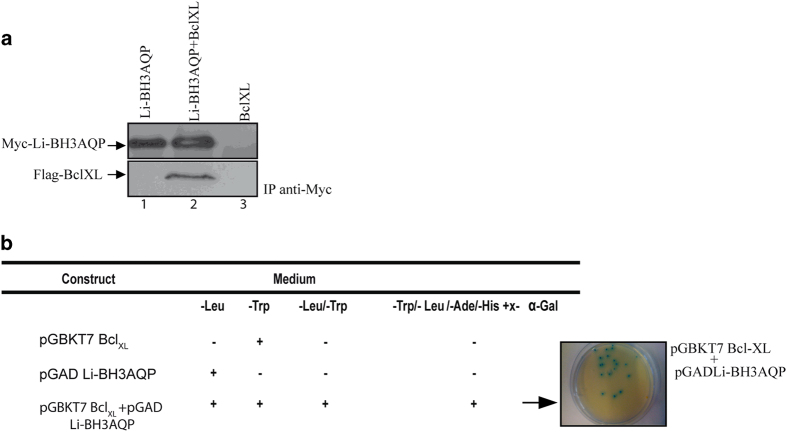
Li-BH3AQP associates with Bcl-X_L_. (**a**) Myc-Li-BH3AQP and Flag-Bcl-X_L_ co-immunoprecipitation using an anti-Myc antibody in HEK293T whole-cell extracts. Immunoprecipitated fractions were blotted against an anti-Flag antibody to detect Flag-Bcl-X_L_. (**b**) Growth of the AH109 yeast strain transfected with *pGBKT7-Bcl-X_L_*, *pGADT7-Li-BH3AQP* or co-transfected with both plasmids. Yeast growth was evaluated in different selective culture medium: (i) −Leu, (ii) −Trp, (iii) −Leu/−Trp and (iv) −Leu/−Trp/−Ade/−His+x-*α*-Gal.

To assess whether Li-BH3AQP might be able to bind to antiapoptotic members of the Bcl-2 family, we coexpressed a Myc-tagged version of Li-BH3AQP and a Flag-tagged version of human Bcl-X_L_ in HEK293T cells. At 24 h after transfection, cells were lysed and expression of these two proteins was confirmed by western blot. Anti-Myc antibodies were used to immunoprecipitate Myc-tagged Li-BH3AQP and the presence of Flag-tagged Bcl-X_L_ was confirmed by western blot with anti-Flag antibodies. The results shown in Figure 4a demonstrate that these two proteins bind to each other inside HEK293T cells.

The interaction between Li-BH3AQP and Bcl-X_L_ was further demonstrated by the yeast two-hybrid system using Bcl-X_L_ (bound to the DNA-binding domain of GAL4) as the ‘bait’ and Li-BH3AQP (bound to the activation domain of GAL4) as the ‘prey’. As shown in Figure 4b, *Saccharomyces cerevisiae* cells transfected with the *pGBKT7-Bcl-X*_*L*_ construct grow in the absence of tryptophan (−Trp) but not in the other three restrictive conditions (−Leu; −Leu/−Trp or −Leu/−Trp/−Ade/−His+x-*α*-Gal). Cells transfected with the *pGADT7-Li-BH3AQP* construct grow in the absence of leucine (−Leu) but not in any other condition. Owing to the interaction of both proteins in the nuclei, cells transfected with both constructions grow in the absence of adenine or histidine. These cells were also able to hydrolyze x-*α*-Gal.

### Reduced viability of HeLa cells expressing Li-BH3AQP

**Figure 5:**
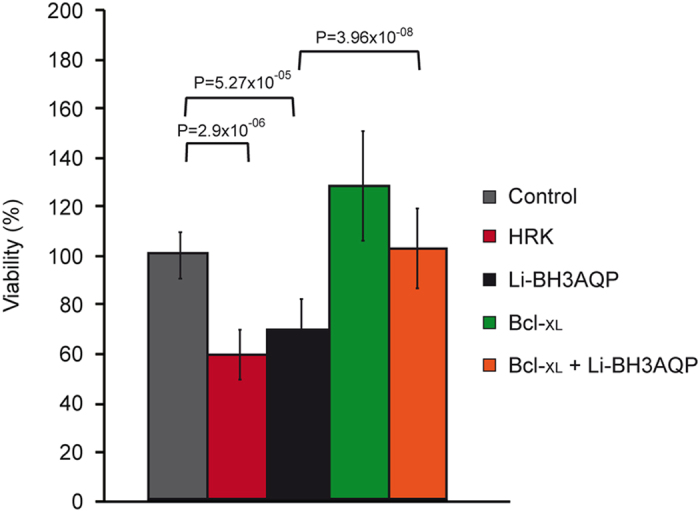
Li-BH3AQP promotes HeLa cell death. Cell viability was determined by the crystal violet method 48 h after transfection of HeLa cells with 500 ng of *pCDNA3* (control), *pCDNA3Hrk* (HRK), *pCDNA3Myc-Li-BH3AQP* (Li-BH3AQP), *pCDNA3Flag-Bcl-X*_*L*_ (Bcl-X_L_) and *pCDNA3Flag-Bcl-X*_*L*_*+pCDNA3Myc-Li-BH3AQP* (Bcl-X_L_+Li-BH3AQP). Mean values of the samples are displayed in bar graphs. Error bars represent the 99.0% confidence intervals calculated for each mean; *n*=12.

It has been shown previously that transfection of human cells with DNA constructions that induce overexpression of proapoptotic Bcl-2 family members causes a marked reduction in the number of viable cells.^35^ Accordingly, to test whether Li-BH3AQP may behave as a proapoptotic protein, the viability of HeLa cells transfected with a vector that allows expression of Li-BH3AQP (*pCDNA3Myc-Li-BH3AQP*) was assayed and compared with that of cells transfected with the empty vector pCDNA3 (Figure 5). As a control, cells were also transfected with a sequence encoding the human Hrk proapoptotic protein (*pCDNA3Hrk*). At 48 h after transfection, a significant reduction in the number of viable cells was observed for both Hrk- and Li-BH3AQP-expressing cells. Coexpression of Bcl-X_L_ (*pCDNA3Myc-Li-BH3AQP*+*pCDNA3Flag-Bcl-X_L_*) restored the viability of Li-BH3AQP-expressing cells to values similar to that of the pCDNA3 control. These results support the notion that Li-BH3AQP behaves as a proapoptotic protein in HeLa cells.

### Overexpression of Li-BH3AQP in *L. infantum* cells

**Figure 6:**
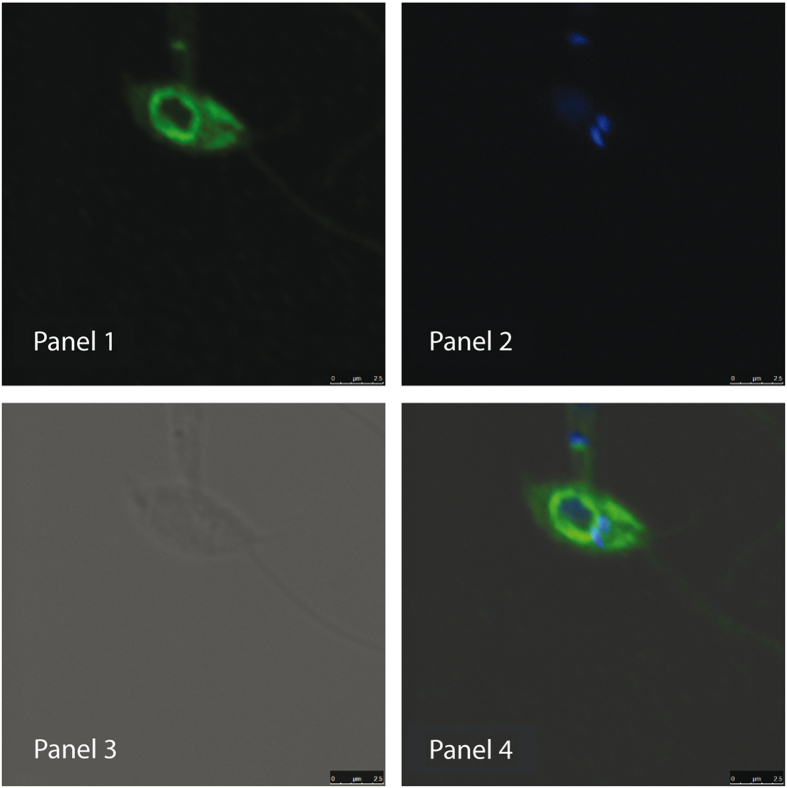
Cellular localization of Li-BH3AQP. (Panel 1) Confocal microscopy of Li-BH3AQP-overexpressing *L. infantum* promastigotes stained with anti-HA antibodies; (panel 2) DAPI (4',6-diamidino-2-phenylindole) staining; (panel 3) differential interference contrast (DIC); (panel 4) merged micrograph.

To obtain further insight into the activity of this protein in *Leishmania* parasites, we fused the sequence coding for an HA epitope at the 3′ end of *Li-BH3AQP* and then cloned it in the pIRmcs-3 (−) vector. Parasites transfected with this construction were selected in the presence of nourseothricin. No significant changes in growth rates were observed between parasites expressing the protein and those transfected with the empty vector. Similar results were obtained when expression of the protein was suddenly induced with tetracycline in *L. tarentolae* parasites (data not shown). The nourseothricin-resistant *L. infantum* promastigotes were incubated with antibodies against the HA epitope to detect the cellular localization of the tagged protein. According to the results shown in Figure 6, *Li-BH3AQP* is located in the perinuclear region and extends to some compartments inside the cells. This finding probably reflects its localization in the secretory pathway, as expected because of the predicted presence of multiple transmembrane regions.

The effect of overexpressing Li-BH3AQP in *L. infantum* promastigotes was evaluated in three different conditions: (i) incubation with staurosporine, (ii) nutrient deprivation and (iii) hypotonic stress. To estimate the relevance of the BH3 domain, wild-type (WT) parasites were also transfected with a vector containing the sequence encoding a Li-BH3AQP variant in which residues 287 and 292 belonging to the BH3 domain (positions 5 and 10 in the PS01259 motif) were replaced by Lys (Li-BH3AQP^L287K/D292K^). After selection with nourseothricin, these parasites were likewise incubated in the three conditions described above and survival rates were compared with those of parasites expressing WT Li-BH3AQP and with control parasites transfected with the empty vector.

**Figure 7:**
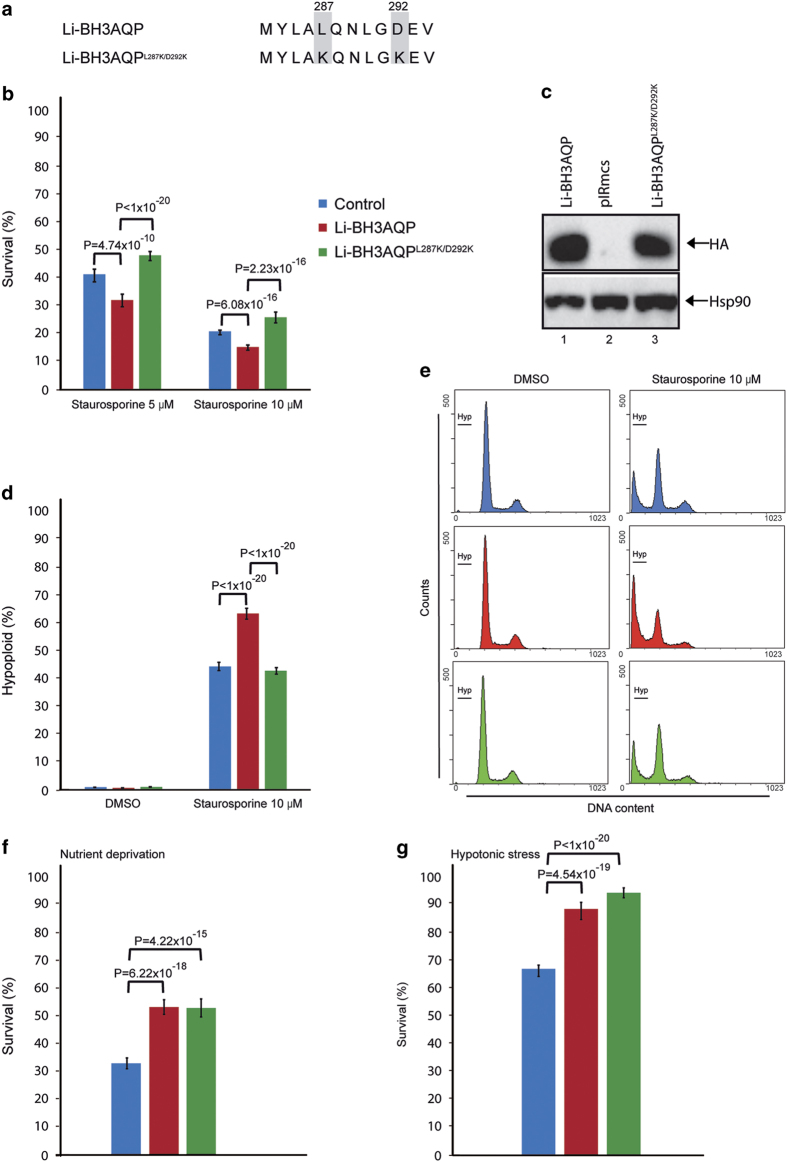
Dual role of Li-BH3AQP on *L. infantum *promastigote survival under diverse death stimuli. Non-transfected parasites (control) and parasites overexpressing WT Li-BH3AQP or Li-BH3AQP^L287K/D292K^ were incubated under different stressing conditions. Mean values of the samples are displayed in bar graphs. Error bars represent the 99.0% confidence intervals calculated for each mean. Cell viability was determined in all cases by flow cytometry using the PI exclusion method. (**a**) Amino-acid sequence alignment of the Li-BH3AQP native BH3 domain and the BH3^L287K/D292K^ mutant. (**b**) Staurosporine treatment. Survival rates were determined under staurosporine concentrations of 5 and 10 *μ*M. One percent DMSO was used as vehicle; *n*=24. (**c**) Western blot analysis of *L. infantum* promastigotes transfected with *pIRmcs-Li-BH3AQP* (Li-BH3AQP), with the empty vector (pIRmcs), or with *pIRmcs-Li-BH3AQP*^L287K/D292K^ (Li-BH3AQP^L287K/D292K^). Anti-HA antibodies were used to detect the overexpressed proteins. Anti-Hsp90 antibodies were used as the loading control. (**d**) Quantification of hypoploid cells after 10 *μ*M staurosporine treatment; *n*=24. (**e**) DNA content of control and staurosporine-treated parasites analyzed by flow cytometry. The marker shows the hypoploid population. (**f**) Nutrient deprivation. Survival rates of parasites incubated for 24 h in phosphate-buffered saline (PBS). Percentages are normalized to parasites growing in complete medium; *n*=18. (**g**) Hypotonic stress. Survival rates were determined after incubation of the parasites for 3.5 h in water-diluted 50% PBS. Rates are normalized to parasites incubated in 100% PBS; *n*=24 except for control parasites in which one data point was excluded from the statistical analysis (*n*=23).

Compared with control parasites, those overexpressing WT Li-BH3AQP displayed a significant reduction in the number of live promastigotes after staurosporine treatment at the two concentrations assayed (Figure 7b). In contrast, death rates similar to those of the control were observed in those parasites expressing Li-BH3AQP^L287K/D292K^, that is, the variant in which two key hydrophobic residues have been replaced with lysine. This finding strongly supports the relevance of the BH3 domain in the prodeath effect of Li-BH3AQP. Cell death could also be correlated with the percentages of hypoploid cells in the three populations. Following incubation with 10 *μ*M staurosporine, the percentage of hypoploid cells increased from 1 to 60% in parasites overexpressing WT Li-BH3AQP, whereas in control and Li-BH3AQP^L287K/D292K^-overexpressing parasites it was below 43% (Figures 7d and e). To ensure that the different behavior in the parasites that overexpress WT Li-BH3AQP is not restricted to staurosporine treatment, we analyzed survival rates in response to 24 h of incubation with antimycin A. The results obtained for this drug are equivalent to those described for staurosporine (Supplementary Figure 2).

The same three populations of parasites were then incubated for 24 h in PBS to evaluate their behavior under nutrient-deprived conditions. Compared with controls, parasites overexpressing any of the two versions of the protein were partially protected against death, as indicated by the significant increase in the number of viable cells (Figure 7f). Finally, because of the demonstrated water transport activity of the *L. donovani* ortholog of Li-BH3AQP,^25^ the parasites were kept for 3 h in PBS:water (50:50). Death induction as a consequence of hypotonic stress was diminished in parasites expressing either WT Li-BH3AQP or Li-BH3AQP^L287K/D292K^ (Figure 7g). Taken together, analysis of the viability of the parasites under different stressing conditions reveals that Li-BH3AQP appears to have both prodeath and prosurvival roles.

## Discussion

Our research group and others have previously demonstrated that ectopic expression of antiapoptotic members of the Bcl-2 family of proteins can interfere with death processes in protozoans.^7^ The antiapoptotic activity of these proteins relies mainly on their interaction with other proteins containing a BH3 domain. Accordingly, we initiated a research effort aimed at the identification of proteins containing BH3 domains in *L. infantum*. Even though there is no optimal bioinformatic procedure to reliably predict new BH3-containing proteins,^36^ the PHI-BLAST algorithm identified the MYLALQNLGDEV sequence as the second best match to the PS01259 signature motif in the *Leishmania* genome. Remarkably, this sequence is located at the C terminus of a pore-forming protein, specifically an aquaporin previously named AQP putative (XP_001465642.1), which is strictly conserved in the orthologs of *L. donovani*, *L. major*, *L. mexicana*, *L. braziliensis* and *L. panamensis*. Because of the presence of this putative BH3 domain, we propose to name the *L. infantum* protein Li-BH3AQP. Even though our search in genome databases from trypanosomatids produced a few other interesting candidates containing putative BH3 motifs, we focused on Li-BH3AQP because of its ability to generate pores in plasma membranes. The results presented in Figure 4a demonstrate co-immunoprecipitation of Li-BH3AQP and Bcl-X_L_ in HEK293T cells. This interaction was further supported in the two-hybrid system by the observed growth of *S. cerevisiae* cells transfected with *pGBKT7-Bcl-X_L_* and *pGADT7-Li-BH3AQP* in a reporter medium lacking histidine and adenine. Moreover, HeLa cells expressing Li-BH3AQP showed a marked reduction in viability 24 h after transfection, which was abrogated by coexpression of Bcl-X_L_ (Figure 5). In contrast, overexpression of Li-BH3AQP in *Leishmania* promastigotes did not cause any reduction in viability under normal growth conditions. When parasites were challenged under different stressing conditions, either prodeath or prolife effects were detected depending on the nature of the stimulus. Overexpression of this protein sensitizes promastigotes to staurosporine or antimycin A, but is protective against nutrient withdrawal or hypotonic stress. Mutation of key residues in the BH3 domain abolishes the prodeath effect observed after treatment with any of these drugs but does not affect its prolife role. There is significant evidence to support an evolutionary process whereby key regulators of cell death in higher eukaryotes also contribute to vital cellular functions, for example cell cycle regulation,^37^ DNA damage responses,^38,39^ redox status,^40^ glucose or energy metabolism,^41^ ER physiology,^42^ and mitochondrial morphology and homeostasis.^43,44^ In all of these examples, BH3-containing proteins act as specialized stress sentinels that enable constant homeostatic quality control.

In common with Ybh3p from *Saccharomyces*,^24^ Li-BH3AQP is likely to be a multimembrane-spanning protein that harbors a BH3 domain at the end of the terminal transmembrane segment. This particular location and the absence of the last three amino acids in the consensus BH3 sequence of both Ybh3p and Li-BH3AQP (Figure 1b) are unprecedented features in BH3-containing proteins. It has been argued that the BH3 sequence in Ybh3p lacks conservation in orthologous proteins,^36^ but this is not the case for the motif in Li-BH3AQP, which is present in the sequences of *L. donovani*, *L. major*, *L. mexicana*, *L. braziliensis* and *L. panamensis* orthologs. Both the yeast and the *Leishmania* BH3-containing proteins bind to Bcl-X_L_ even though Bcl-X_L_ orthologs have not been found in any of these two species. This has been considered a relevant argument against the functional relevance of these BH3 motifs. However, as shown for the vaccinia virus protein A49, the 3D structural characteristics of Bcl-2 family members can also be found in proteins with sequence identity as low as 8%.^45^ Therefore, proteins able to interact with BH3 domains may still be found in non-metazoan cells. Given that *Leishmania* parasites are intracellular and that protein migration from the parasites to the cell cytoplasm has already been demonstrated,^46^ it is also plausible that Li-BH3AQP might interact with antiapoptotic members of the host cells to interfere in their apoptotic programs. Experiments to analyze translocation of Li-BH3AQP from the parasites to the host cell are currently in progress.

In summary, our results demonstrate that: (i) a short amino-acid stretch in the C-terminal region of Li-BH3AQP displays structural properties that can account for the specific binding to Bcl-X_L_, (ii) Li-BH3AQP interacts with Bcl-X_L_ both in yeast and mammalian cells, (iii) ectopic expression of Li-BH3AQP in HeLa cells causes a reduction in cell viability that can be reverted by overexpression of Bcl-X_L_ and (iv) the BH3 domain in Li-BH3AQP is responsible for the reduced viability of *L. infantum* promastigotes treated with staurosporine or antimycin A.

Taken together, our results strongly suggest that Li-BH3AQP could well be the first non-enzymatic molecule involved in the cell death pathway in trypanosomatids. Moreover, our data support the view that protein–protein interactions involving BH3 domains might have appeared much earlier than expected during evolution even though they might not necessarily have been originally dedicated to orchestrate death processes.

## Materials and Methods

### Bioinformatics analyses

#### Bioinformatics analysis and molecular modeling

The Li-BH3AQP DNA sequence was obtained from the kinetoplastid database http://tritrypdb.org (DNA i.d. XP_001465642.1).^25^ Any other DNA or protein *s*equences were obtained from the National Center for Biotechnology Information (NCBI). The PHI-BLAST algorithm was applied to search for putative BH3-containing sequences in the *Leishmania* genus. The central region (residues 5–12) of the Bcl-2 family BH3 motif signature (L-[KARQ]-x-[IVAL]-G-D-[DESG]-[LIMFV] (Prosite entry PS01259) was used as the query sequence. After identification of a putative BH3 motif in the *Leishmania* genus, sequence alignments against other BH3-containing proteins were performed using the Clustal W software (Conway Institute UCD, Dublin, Ireland).^47^

Phyre2.0^48^ and Swiss-Model^49^ servers were used for the homology modeling of Li-BH3AQP (residues 1–294, UNIPROT i.d. A4I001). The crystal structures of Pp-Aqy1 (PDB entry 2W2E, 26% i.d.; Fischer *et al*.^28^) and Mm-AqpM (PDB entry 2F2B, 29% i.d.; Lee *et al*.^29^) were used as templates. Water molecules shown in Figure 2 were taken from 2F2B and energy was minimized using sander (AmberTools15; AMBER) inside a channel of one Li-BH3AQP monomer. Affinity maps on the surface of Bcl-X_L_ (PDB i.d. 1PQ1)^34^ were created using our in-house tool cGRILL.^32^ All molecules were visualized in PyMOL (DeLano, W. PyMOL, v.1.3).

### Reagents

All reagents were obtained from Sigma‐Aldrich (Saint Louis, MO, USA). Restriction enzymes, DNA polymerases, T4 DNA ligases, DNAse and alkaline phosphatase were obtained from Takara (Kusatsu, Japan), ThermoFisher (Waltham, MA, USA), Roche (Penzberg, Germany) and New England Biolabs (Ipswich, MA, USA). Gel purification and Maxi and miniprep kits were obtained from Qiagen (Hilden, Germany).

### Cells and culture conditions

HeLa cells were kindly provided by Dr. Muñoz (Instituto de Investigaciones Biomédicas Alberto Sols CSIC-UAM, Madrid, Spain) and authenticated using the GenePrint 10 System (Promega Corporation, Fitchburg, WI, USA; 100% match with the reference strain). Cells were maintained in Dulbecco’s modified Eagle’s medium (DMEM; Sigma-Aldrich) supplemented with 10% fetal calf serum (FCS) (Gibco BRL Life Technologies, Paisley, UK), 100 U/ml penicillin (Gibco BRL Life Technologies), 100 *μ*g/ml streptomycin (Gibco BRL Life Technologies) and 10 mM HEPES at 37 °C and 5% CO_2_. Cells were tested for mycoplasma contamination.

HEK293T cells were kindly provided by Dr. Alonso (Centro de Biología Molecular ‘Severo Ochoa’ (CBMSO), Madrid, Spain) and maintained as monolayer cultures in 75-cm^2^ tissue culture flasks in DMEM supplemented with 10% fetal bovine serum, 100 U/ml penicillin, 100 *μ*g/ml streptomycin and 25 *μ*g/ml amphotericin, in a humidified 5% CO_2_/95% air incubator at 37 °C. This cell line was also authenticated using the GenePrint 10 System (Promega Corporation, Fitchburg, WI, USA; 94% match with the reference strain). Cells were tested for mycoplasma contamination.

*L. infantum* promastigotes (M/CAN/ES/96/BCN150 MON-1), kindly provided by Dr. Alonso (CBMSO-Universidad Autónoma, Madrid, Spain), were grown in RPMI-1640 medium (Gibco, Paisley, UK) supplemented with 10% heat-inactivated FCS, 100 U/ml penicillin, 100 *μ*g/ml streptomycin and 25 mM HEPES at 26 °C. Recombinant parasites were obtained by electroporation with the pIRmcs-3 plasmid containing the coding sequence of Li-BH3AQP or its mutated version Li-BH3AQP^L287K/D292K^. The parasites were selected in nourseothricin-containing medium during 1 week and used for further experiments.

*S. cerevisiae* strain AH109 (Clontech, Mountain View, CA, USA), genotype: MATa, trp1-901, leu2-3, 112, ura3-52, his3-200, gal4D, gal80D, LYS2:: GAL1_UAS_-GAL1_TATA_-HIS3, GAL2_UAS_-GAL2_TATA_-ADE2,URA3:: MEL1_UAS_-MEL1 _TATA_-lacZ was grown in the YPD medium (10 g yeast extract, 20 g bacto peptone and 20 g dextrose (glucose) at 30 °C). The cells were subsequently transformed by heat shock with different plasmids containing the sequences of human Bcl-X_L_ or Li-BH3AQP. The recombinant cells were maintained under selective pressure in solid agar medium.

### HeLa cells transfection and viability

Transiently lipofectamine-transfected cells were used to study the overexpression effects of Li-BH3AQP, as well as human Bcl-X_L_ and HRK proteins. Five hundred microliters of a 50 000 cells per ml suspension were placed in 24-well cell culture plates. After 24 h incubation, cell culture was removed and 250 *μ*l of a mixture of Lipofectamine (ThermoFisher), Optimem (ThermoFisher) and DNA (500 ng) was added to each well. Two hundred and fifty microliters of DMEM without antibiotics were subsequently added. Media were removed after 24 h and cells were incubated for 24 additional hours with complete medium. HeLa cell viability was determined by the crystal violet assay. Briefly, cells were stained with crystal violet 48 h after transfection with DNA constructions that allow expression of Li-BH3AQP, HRK or Bcl-X_L_. Color intensity was quantified using a spectrophotometer at 570 nm.

### Protein electrophoresis and immunodetection

A total of 10×10^6^ parasites were lysed in 100 *μ*l of RIPA buffer and subsequently precipitated with four volumes of cold acetone at −20 °C during 60 min. Precipitated protein was centrifuged at 18 000×*g* for 30 min and the protein pellet was solubilized in 8 M urea. The protein concentration was determined by the BCA protein assay system (ThermoFisher). Each sample was boiled in Laemmli buffer before using it for SDS-PAGE analysis in 10% acrylamide gels. For western blot analysis, proteins were transferred from the gels to a PVDF membrane in transfer buffer (25 mM Tris-HCl, 192 mM glycine, 20% methanol, 0.02% SDS, pH 8.3). The membranes were first incubated for 1 h in a blocking solution consisting of TBS-T buffer (150 mM NaCl, 10 mM Tris, 0.1% Tween, pH 8) supplemented with 5% of bovine serum albumin (Sigma-Aldrich) and then with anti-HA (1:250; Sigma‐Aldrich; cat. no. H6908) or anti-Hsp90 (1:2500; Dr. Clos laboratory, Bernhard Nocht Institute for Tropical Medicine, Hamburg, Germany) antibodies during 16 h at 4 °C with shaking. An anti-rabbit (1:2000) antibody conjugated with HRP (SC-2004; Santa Cruz Biotechnology, Dallas, TX, USA) was used as the secondary antibody for HA detection and a goat anti-chicken IgY was used as the secondary antibody for *Leishmania* Hsp90 detection (SC-2428; Santa Cruz Biotechnology). Antibodies were recognized with ECL reagent (Thermo Scientific, Waltham, MA, USA).

### Co-immunoprecipitation

*Bcl-X_L_* and *Li-BH3AQP* genes were cloned into pCDNA3 plasmids fused either with Flag or Myc tags, respectively. HEK293T cells were collected after transient transfection with the different combinations of constructions for 24 h and used for whole-cell extract preparation. Immunoprecipitation was carried out using a resin-conjugated antibody (anti-myc) complex (A7470-1 ml; Sigma, St Louis, MO, USA). After extensive washing steps, the immunoprecipitated fraction was used for western blotting using an anti-Flag antibody to detect Bcl-X_L_ (monoclonal ANTI-FLAG M2, Clone M2; F3165; Sigma‐Aldrich) and an anti-myc antibody to detect *Li-BH3AQ*P (sc40; Santa Cruz Biotechnology, Dallas, Texas, USA).

### Yeast two-hybrid assay

In this assay, human Bcl-X_L_ was used as a bait and Li-BH3AQP as a prey. Briefly, when both bait and prey proteins have a physical interaction, a Gal4 transcription factor activates transcription of four reporter genes (*ADE2*, *HIS3*, *LacZ* and *MEL1*) allowing cellular growth in a minimal medium lacking histidine and adenine. Furthermore, the *MEL1* gene encodes for *α*-galactosidase that allows transformation of the chromogenic substrate x-*α*-Gal into a blue product. Bcl-X_L_ was cloned in the BpGBK-T7 plasmid that allows cell growth in a medium lacking the Trp. Li-BH3AQP was cloned in the pGADT7 plasmid that allows cell growth in a medium lacking the Leu. To confirm expression of both proteins, a medium lacking Trp and Leu (−Trp/−Leu) was used to select the cotransformed cells. Finally, some of these colonies were plated into a agar medium lacking Trp, Leu, His and Ade in the presence of the chromogenic substrate x-*α*-Gal.

### Confocal microscopy

Logarithmic growth phase parasites were fixed using a solution containing 2% paraformaldehyde and permeabilised with 0.1% Triton X-100. Anti-HA (Sigma‐Aldrich; cat. no. H6908; 1:50) antibody was used to determine the localization of Li-BH3AQP and DAPI was used to visualize the nuclei. Alexa Fluor 488 anti-rabbit antibody was used as secondary antibody (Life Technologies). All preparations were mounted using ProLong Gold Antifade Reagent (Life Technologies) and analyzed using a Leica TCS SL Microscope (Leica Microsystems GmbH, Wetzlar, Germany).

### Drug treatment of the parasites

Staurosporine and antimycin A treatments of promastigotes were performed during the logarithmic growth phase at the drug concentrations indicated in the figures for 24 h. *L. infantum* promastigotes were diluted in 10 ml of cell culture medium at 2×10^6^ cells per ml. After 24 h, parasites were diluted again to 2×10^6^ cells per ml and 200 *μ*l aliquots were placed in 96-well cell culture plates. Two microliters of a stock solution of staurosporine or 3 *μ*l of a stock solution of antimycin A were added to each well. Equivalent volumes of DMSO were added to controls. After 24 h of incubation at 26 °C, parasite viability was evaluated by flow cytometry by the propidium iodide (PI) exclusion method. Briefly, treated parasites were stained for 10 min with 10 *μ*g/ml PI. The number of PI− parasites was determined in a Beckman Coulter FC500 flow cytometer(Brea, CA, USA).

### Nutrient deprivation

*L. infantum* promastigotes were diluted in 10 ml of cell culture medium at 2×10^6^ cells per ml. After 24 h, cells were counted and, for each sample, 600 000 parasites were centrifuged at 1000×*g* for 5 min. Pellets were resuspended in 300 *μ*l of cell culture medium or PBS and 200 *μ*l (2×10^6^ promastigotes per ml) were dispensed in 96-well cell culture plates. After 24 h of incubation, parasite viability was evaluated by flow cytometry by the PI exclusion method.

### Hypotonic stress

*L. infantum* promastigotes were diluted in 10 ml of cell culture medium at 2×10^6^ cells per ml. After 24 h, cells were counted, centrifuged and resuspended at 32×10^6^ parasites per ml in PBS. Fifty microliters of this suspension (1.6×10^6^ parasites) were placed in 96-well cell culture plates and 150 *μ*l of 33% PBS diluted in sterilized H_2_O was added to reach a final PBS concentration of 50%. After 3.5 h of incubation, parasite viability was evaluated by flow cytometry by the PI exclusion method.

### DNA content analysis by flow cytometry

*L. infantum* promastigotes were diluted in 10 ml of cell culture medium at 2×10^6^ cells per ml. After 24 h, parasites were diluted again to 2×10^6^ cells per ml and 2 ml aliquots were placed in wells of 24-well cell culture plates. Twenty microliters of a stock solution of staurosporine (1 mM) were added to each well. Parasites were centrifuged at 1000×*g* for 5 min, the pellet was resuspended in 100 *μ*l of ice-cold PBS and 700 *μ*l of 80% ethanol at −20 °C were added and then parasites were incubated overnight at −20 °C. After incubation, the parasites were washed with 800 *μ*l of PBS, pelleted at 1000×*g* and then resuspended in 400 *μ*l of PBS/10 *μ*g/ml PI/50 *μ*g/ml RNAse and incubated for 30 min at 37 °C. The parasites were then analyzed for PI fluorescence in a Beckman Coulter FC500 flow cytometer (Brea, CA, USA).

### Statistical analysis

Standardized skewness and standardized kurtosis were analyzed to determine whether the samples come from normal distributions. To ensure normality, values of these statistics were always tested to be within the range of −2 to +2. Kolmogorov–Smirnov test was also run to determine whether the samples could be adequately modeled by a normal distribution.

Once the normal distribution of data was checked, the Welch’s unequal variances *t*-test was used to compare means of different samples.^50,51^ This test is designed for normally distributed samples that might have unequal variances. The Excel T.Test function with the specific parameters for a two-tailed analysis of heteroskedastic independent samples was used to compute *P*-values and decide if the null hypothesis (mean 1=mean 2) can be rejected. Even though it was not needed, variances were checked for similarity in all samples. The number of independent replicates (*n*) was adjusted to be the highest possible without increasing the risk of human errors. The *n* value is indicated in the figure legends. Experiments were carried out in three different days and the replicates were equally distributed among them.

Mean values of the samples are displayed in bar graphs. Error bars represent the 99.0% confidence intervals calculated for each mean. Data points out from the range of the mean±3 S.D. were excluded from the analysis. Among all the samples analyzed, only one data point obtained during the hypotonic stress was found to be out of this range and, consequently, was omitted. This fact is indicated in the legend to Figure 7g.

Groups of numerical data are also depicted in standard box-and-whisker plots in the Supplementary Material Section (Supplementary Figure S3). Statgraphics centurion XVII program was used for calculation of the mean confidence intervals and for boxplot representations.
