# Supplementary figures and images for: A functional BH3 domain in an aquaporin from Leishmania infantum
Source: Cell Death Discov. 2016 Jul 4;2:16043–. doi: 10.1038/cddiscovery.2016.43 (PMC4979448; doi:10.1038/cddiscovery.2016.43)

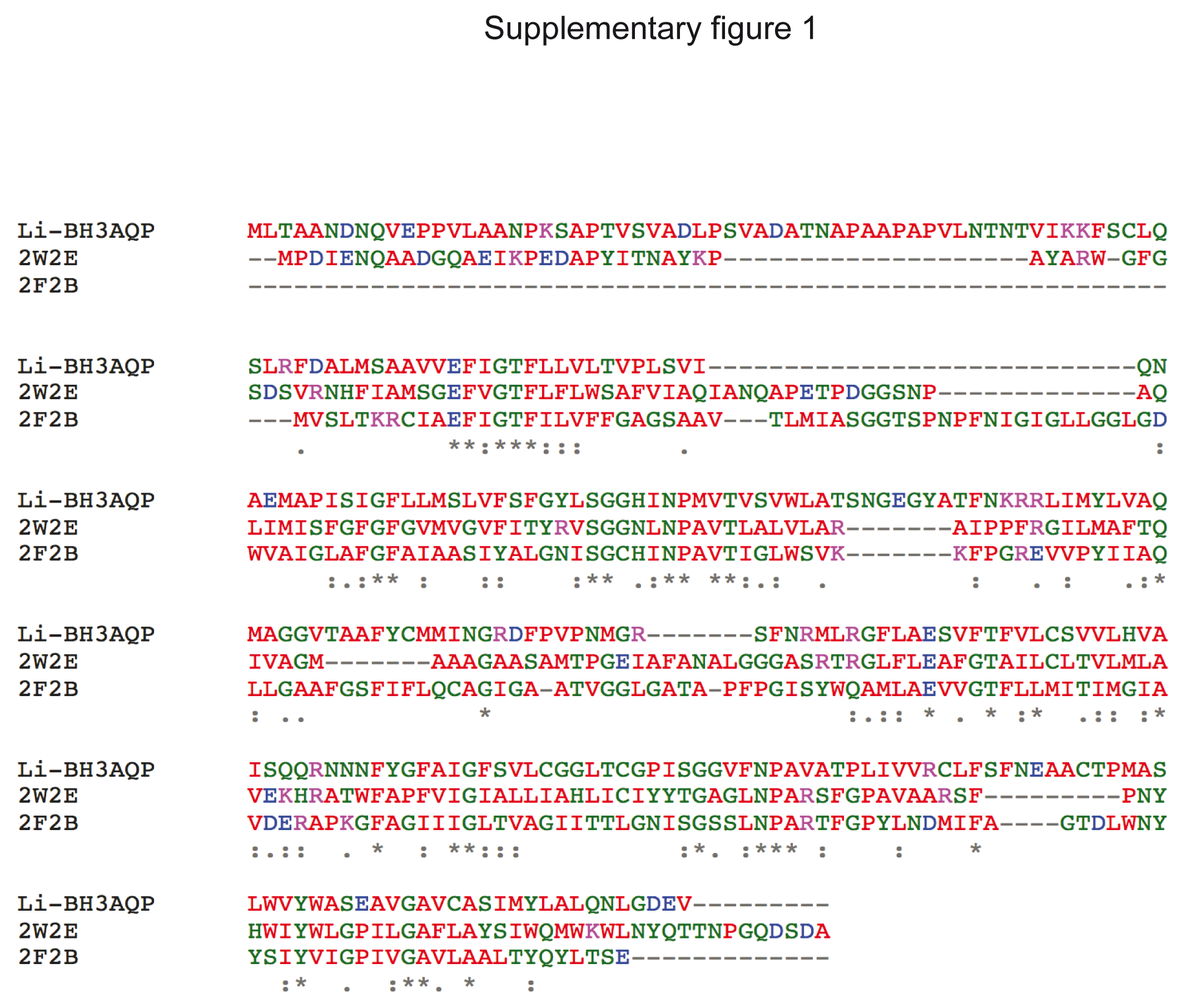

Supplement: Supplementary Figure 1 [file cddiscovery201643-s1.tiff]

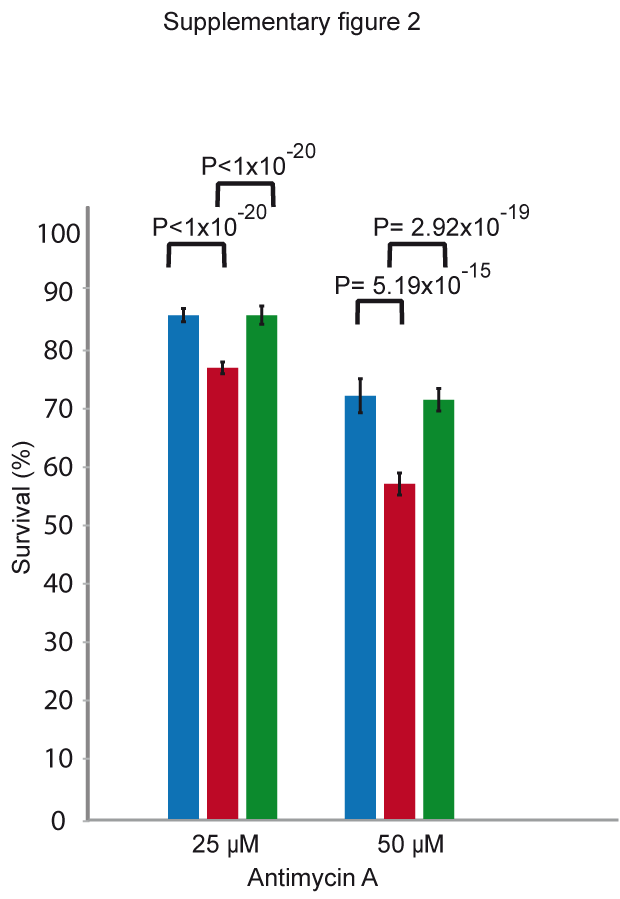

Supplement: Supplementary Figure 2 [file cddiscovery201643-s2.tiff]

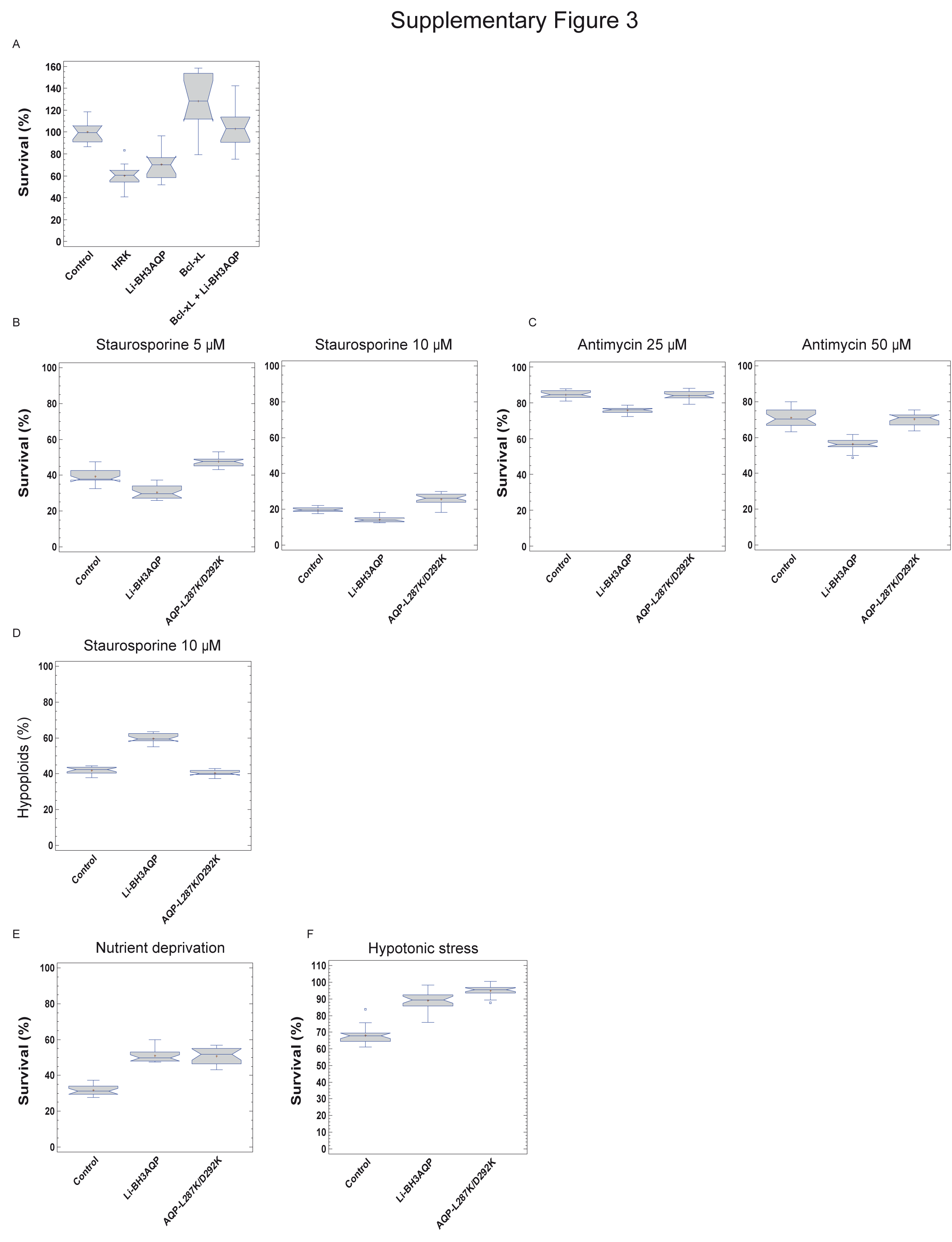

Supplement: Supplementary Figure 3 [file cddiscovery201643-s3.tiff]
